# A Medical Mecca

**Published:** 1891-10

**Authors:** 


### A MEDICAL MECCA.

The City of St. Louis will be entitled to the banner as a center of attraction for medical men during the week beginning October 12, 1891. The Mississippi Valley Medical Association will hold its seventeenth annual meeting there October 14th, 15th and 16th ; the Association of American Medical Editors will also convene during the same week ; the Board of Trustees of the American Medical Association will hold an adjourned meeting at that time ; and, finally, the Executive Committee of the Inter-Continental Medical Congress will convene for the organization of the Congress during the same eventful week. This latter will be a most important meeting, and the chairman, Dr. Charles A. L. Reed, of Cincinnati, is desirous that all interested shall meet and confer with the committee, with a view to the perfection of this most important medical organization. Surely the attractions are many for physicians at St. Louis during the mid-October season.

